# A call for linguistic and culturally congruent family-centred early hearing detection and intervention programmes in South Africa

**DOI:** 10.4102/sajcd.v71i1.992

**Published:** 2024-03-19

**Authors:** Ntsako P. Maluleke

**Affiliations:** 1 Department of Audiology, Faculty of Humanities, University of the Witwatersrand, Johannesburg, South Africa

**Keywords:** early intervention, EHDI, family-centred early intervention, FCEI, hearing impairment, hearing screening

## Abstract

Early Hearing Detection and Intervention (EHDI) programmes are recognised as the standard of care for newborns and infants presenting with hearing impairment, globally. However, widespread implementation of these programmes is far from being realised and faces numerous challenges within the South African context. The United Nations’ sustainable development goal 3.8 and South Africa’s national development plan seek to achieve equitable access to healthcare service, including EHDI. However, healthcare access is a complex concept which encompasses the dimensions: availability, affordability, acceptability and accommodation in healthcare. South Africa has made great progress towards universal implementation of EHDI programmes. Despite this progress, availability and affordability of these programmes are limited and their acceptability has received limited research focus in this context. Furthermore, accommodation of caregivers, as co-drivers of EHDI programmes and ensuring that EHDI programmes are linguistically and culturally congruent have also been overlooked within the South African context.

**Contribution:**

Increased robust efforts in improving access through availability and affordability of EHDI programmes are warranted in South Africa. However, improving access to these programmes through availability and affordability initiatives alone will not result in a pragmatic improvement in their accessibility. Acceptability of these programmes and accommodations such as involving caregivers and family members of children with hearing impairment as equal partners in EHDI programmes and being cognisant of their linguistic and cultural needs must be considered.

Early hearing detection and intervention (EHDI) programmes are recognised as the standard of care for newborns and infants presenting with hearing impairment (Health Professions Council of South Africa [HPCSA], 2018; Naidoo & Khan, 2022). Early hearing detection and intervention encompasses the earliest possible identification, diagnosis and intervention for these children, to ensure that they can communicate effectively and develop to their maximum potential (HPCSA, 2018; Kanji & Khoza-Shangase, 2021; Maluleke & Khoza-Shangase, 2023). These programmes are significant within the South African context, where the prevalence of a permanent hearing impairment is reported to be 3–6 per 1000 births in the public sector (Kanji & Khoza-Shangase, 2019; Khoza-Shangase & Mophosho, 2018; Khoza-Shangase et al., 2017). Despite the high prevalence of infant hearing impairment and global and national healthcare reform initiatives towards equitable access to healthcare, widespread implementation of EHDI programmes is far from being realised in South Africa and faces numerous challenges (Khoza-Shangase, 2019; United Nations General Assembly, 2015; World Health Organization [WHO], 2021). These challenges include an over-burdened public healthcare sector, quadruple burden of disease challenges, social determinants of health challenges, resource limitations, poor knowledge and awareness of EHDI among healthcare professionals (HCPs) and caregivers, and a lack of government mandate for EHDI (Kanji & Khoza-Shangase, 2021; Maluleke et al., 2023a; Naidoo & Khan, 2022; Petrocchi-Bartal et al., 2021; WHO, 2021).

Consistent with the United Nations’ sustainable development goal 3.8, South Africa’s national development plan aims to provide quality healthcare that is universal and equitable to all its citizens through the National Health Insurance (NHI) Bill (Department of Health, 2017; National Planning Commission, 2013; United Nations General Assembly, 2015). However, healthcare access is a complex concept, with numerous frameworks (Cu et al., 2021; Levesque et al., 2013; Ryvicker, 2019). Consensus among the various frameworks and authors is that access to healthcare comprises four interdependent dimensions: (1) availability (proximity of the healthcare facility [HCF], and the whether the facility has the necessary technology and personnel to meet the patient’s needs); (2) affordability (direct and indirect costs associated with the use of healthcare services and whether or not the patient is able to pay for these services); (3) acceptability (patient’s perception of the services’ appropriateness and effectiveness in addressing their health concern, and suitability to their lifestyle and convenience); and (4) accommodation (the services’ ability to meet the patients’ needs for care, preferences and constraints) (Burger & Christian, 2018; Cu et al., 2021; Gordon et al., 2020; Levesque et al., 2013; Sekhon et al., 2017; Wyszewianskin & McLaughlin, 2002).

A review of available literature on EHDI in South Africa highlights ongoing efforts towards universal implementation of EHDI programmes, considering the dimensions of access (Hussein et al., 2018; Kanji, 2016; Khan et al., 2018; Naidoo & Khan, 2022). Current newborn hearing screening programmes are conducted: (1) at both private and public HCFs, including primary healthcare clinics, (2) by audiologists or nurses, healthcare workers and volunteers in what is referred to as task-shifting (or task sharing, upskilling or role release), (3) using otoacoustic emissions, automated auditory brainstem response or mobile health (mHealth) technologies, and (4) for free at public HCFs, or out-of-pocket at private HCFs (Gordon et al., 2020; Hussein et al., 2018; Khoza-Shangase, 2021; Petrocchi-Bartal et al., 2021; Storbeck & Young, 2016; Wilford et al., 2018). While early intervention services are provided at private and public HCFs, the child’s home or in specialised pre-schools and primary schools (Kanji, 2021), availability and affordability of EHDI programmes are limited, resulting in late diagnosis and intervention for children with hearing impairment (Ehlert & Coetzer, 2020; Hussein et al., 2018; Khoza-Shangase, 2019; Swanepoel & Clark, 2019). Human resource capacity challenges and the high cost associated with EHDI services, amplification devices and accessories within the private sector, as well as transport costs to public HCFs have been identified as barriers to the availability and affordability of EHDI programmes (Hussein et al., 2018; Khan et al., 2018; Khoza-Shangase, 2019; Maluleke et al., 2023a).

*Acceptability* of current EHDI programmes has received limited research focus within the South African context. Maluleke et al. (2023b) was the first study within the South African context to investigate caregivers’ perceptions of the acceptability of EHDI programmes. Patients are more likely to comply with treatment recommendations when intervention is considered to be acceptable, which can have a significant impact on the effectiveness of the treatment and improved outcomes for patients (Sekhon et al., 2017, Mtimkulu et al., 2023). Thus, further exploration of acceptability of EHDI programmes within this context is warranted, especially considering the reported poor follow-up rates (Kanji & Krabbenhoft, 2018). An understanding of the acceptability of current EHDI programmes would ensure that these programmes are tailored and customised according to community needs and preferences, assist in policy formulation and successful implementation of these programmes (Khoza-Shangase, 2022; Mtimkulu et al., 2023; Sekhon et al., 2017).

Another dimension that has been overlooked in clinical practice and research efforts is *accommodation* – the ability of current EHDI programmes to accommodate the patient’s needs of care and the constraints they encounter. Aspects of accommodation that have been overlooked include recognition of caregivers as co-drivers of EHDI programmes, and ensuring that EHDI programmes are linguistically and culturally congruent. Inclusion of caregivers as active partners in the care and decision-making for children with hearing impairment represents a paradigm shift in healthcare (Khoza-Shangase, 2019; Maluleke et al., 2021a, 2021b), and aligns with the HPCSA’s (2018) recommendation that early intervention services following diagnosis of hearing impairment must be family-centred, community-based, and culturally congruent. This is also in line with an Afrocentric ethos of *ubuntu.*

Caregivers and family members of the child with hearing impairment are the ones who are most involved with the child, provide a rich cultural context, and have a greater influence on the child’s development than EHDI personnel who spend small portions of time with the child (Maluleke et al., 2021a; Mantri-Langeveldt et al., 2019; Schlebusch, Samuels & Dada, 2016). Hence, incorporating the family’s routines, language, culture and beliefs in intervention practices is recommended to ensure effective family-centred EHDI (FC-EHDI) programmes (Balton et al., 2019; Khoza-Shangase, 2022; Maluleke et al., 2021a; Schlebusch et al., 2016). Maluleke et al. (2021a) argue that establishing FC-EHDI within the South African context may curtail the access challenges associated with EHDI programmes. Children spend a considerable amount of time with their caregivers and families, making them the most cost-effective system for nurturing the child’s development (Maluleke et al., 2021a). Family-centred EHDI is a collaboration between professionals and the child’s caregivers and addresses the child’s needs within the context of their family. Family-centred EHDI optimises the child’s developmental outcomes by educating, supporting and empowering caregivers and family members of the child with hearing impairment (Iversen et al., 2003; MacKean & Thurston, 2005). When caregivers and families are empowered through linguistically and culturally congruent FC-EHDI programmes, they can optimise their child’s developmental outcomes while ensuring that their child is not distanced from their language and cultural heritage (HPCSA, 2018; Khoza-Shange & Mophosho, 2018; Maluleke et al., 2021a).

Implementing FC-EHDI programmes within this South African context would curtail the language constraints experienced by caregivers when accessing EHDI programmes (Khoza-Shangase, 2019; Maluleke et al., 2023a). English is the predominant language in accessing healthcare in most HCFs, despite South Africa having 12 official languages (Constitution of South Africa, 1996; Parliament of the Republic of South Africa, 2023; The Presidency, 2023). This practice does not respond to the needs of 11 million South Africans receiving healthcare services in English, resulting in poorer health outcomes (Flood & Rohloff, 2018; Khoza-Shangase & Mophosho, 2018; Maluleke et al., 2021a; Maluleke & Khoza-Shangase, 2023; Mophosho, 2018; Mtimkulu et al., 2023; Steinberg et al., 2016). Linguistically and culturally congruent FC-EHDI programmes can be achieved through: (1) conducting EHDI programmes in all of South Africa’s official languages, which would in turn facilitate caregiver participation and engagement, (2) education and training for EHDI personnel about language and cultural competence, and inclusive practices so they can effectively meet the needs of the diverse communities they serve, and (3) collaborating with caregivers, community leaders, support groups, and others, to gain insight into their unique challenges and needs.

Ultimately, improving access to EHDI programmes in South Africa requires pragmatic considerations across the four domains of access. Focusing solely on availability and affordability initiatives, is insufficient to achieve the desired universal access to EHDI programmes. Addressing the needs of caregivers and families, while considering the linguistic and cultural congruence, is vital. Embracing linguistically and culturally congruent FC-EHDI does not only improve acceptability of these programmes but also ensures cost-effective, equitable, efficacious, and high-quality EHDI programmes for infants and children with hearing impairment, as well as their families within the South African context.
